# An Algorithm for Preclinical Diagnosis of Alzheimer's Disease

**DOI:** 10.3389/fnins.2018.00275

**Published:** 2018-04-30

**Authors:** Tapan K. Khan

**Affiliations:** Center for Neurodegenerative Diseases, Blanchette Rockefeller Neurosciences Institute, West Virginia University, Morgantown, WV, United States

**Keywords:** Alzheimer's disease, neuroimaging (anatomic and functional), CSF, diagnosis, differential, preclinical Alzheimer's disease

## Abstract

Almost all Alzheimer's disease (AD) therapeutic trials have failed in recent years. One of the main reasons for failure is due to designing the disease-modifying clinical trials at the advanced stage of the disease when irreversible brain damage has already occurred. Diagnosis of the preclinical stage of AD and therapeutic intervention at this phase, with a perfect target, are key points to slowing the progression of the disease. Various AD biomarkers hold enormous promise for identifying individuals with preclinical AD and predicting the development of AD dementia in the future, but no single AD biomarker has the capability to distinguish the AD preclinical stage. A combination of complimentary AD biomarkers in cerebrospinal fluid (Aβ_42_, tau, and phosphor-tau), non-invasive neuroimaging, and genetic evidence of AD can detect preclinical AD in the *in-vivo* ante mortem brain. Neuroimaging studies have examined region-specific cerebral blood flow (CBF) and microstructural changes in the preclinical AD brain. Functional MRI (fMRI), diffusion tensor imaging (DTI) MRI, arterial spin labeling (ASL) MRI, and advanced PET have potential application in preclinical AD diagnosis. A well-validated simple framework for diagnosis of preclinical AD is urgently needed. This article proposes a comprehensive preclinical AD diagnostic algorithm based on neuroimaging, CSF biomarkers, and genetic markers.

## Introduction

Therapeutic interventions for Alzheimer's disease (AD) will have a better chance of success if initiated at the earliest stage (preclinical), before the synaptic loss and neuronal death occur. Therefore, an effective disease-modifying clinical trial should target the stage before the manifestation of clinical symptoms of memory loss and cognitive impairment. The term preclinical AD is initially described to classify cognitively normal individuals with evidence of amyloid plaques and hyperphosphorylated tau (p-tau) tangles (hallmarks of AD pathology) at time of brain autopsy (Hubbard et al., 1990). This definition of preclinical AD was built on the existing AD mechanistic hypothesis. Several longitudinal studies predicted the conversion of mild cognitive impairment (MCI) to AD when there is amyloid plaques and p-tau tangles in the brain (Petersen et al., 1999, 2001; Grand et al., 2011). In preclinical familial AD cases, Fox et al. (1999) were the first to use serial structural magnetic resonance imaging (MRI) to detect cerebral atrophy in a longitudinal study of asymptomatic individuals (no cognitive impairment) at high risk of the familial AD (autosomal dominant early-onset before 65 years of age). Since that study, several groups have examined the predictive capacity of various brain biomarkers, such as noninvasive neuroimaging modalities, and invasive cerebrospinal fluid (CSF) biomarkers (Aβ_42_, tau, and p-tau). These studies concluded that patients who are positive for various AD biomarkers in the preclinical AD stage are at higher risk of progressing to AD dementia (Mintun et al., 2006; Villemagne et al., 2008; Johnson et al., 2012; Dubois et al., 2016). Non-invasive neuroimaging modalities, such as positron emission tomography (PET), functional MRI (fMRI), diffusion tensor imaging (DTI) MRI, and arterial spin labeling (ASL) MRI hold enormous promise for identifying preclinical AD (Mintun et al., 2006; Mosconi et al., 2006; Villemagne et al., 2008; Johnson et al., 2012; Ewers et al., 2013; Dubois et al., 2016) (Table 2). CSF biomarkers can predict decreasing cognitive ability in studies of conversion of MCI to AD (Frölich et al., 2017) and predicting memory deficit in longitudinal studies of normal individuals (Fagan et al., 2007; Gustafson et al., 2007; Stomrud et al., 2007; Jansen et al., 2015). AD is a multifactorial disease with several genetic biomarkers are found to be involved (http://www.alzgene.org) in the pathogenesis of AD. Those genes increase the predictability of conversion of preclinical AD to MCI and finally AD. In this perspective review, we describe the utility of combining neuroimaging, CSF, and genetic AD biomarker in the diagnosis of preclinical AD and propose a comprehensive preclinical AD diagnostic algorithm.

### Preclinical stage of Alzheimer's disease

The National Institute on Aging and Alzheimer's Association (NIA-AA) (2011) introduced the concept of “preclinical AD” that arises before MCI and advanced stages of AD. The most recent definition of preclinical AD, proposed in a joint meeting of NIA-AA and the International Working Group (IWG) in 2015 (Dubois et al., 2016), is the simplest: preclinical AD starts the day that pathological AD lesions appear without any clinical symptoms. The NIA-AA guideline (2011) divides the progression of AD into distinct phases, taking into account both AD pathobiology and clinical symptoms: preclinical, asymptomatic pre-dementia; symptomatic pre-dementia (MCI); and dementia due to AD (McKhann et al., 2011; Sperling et al., 2011). Before that, preclinical stage AD biomarkers were categorized as Stage 1: amyloidosis by PET and CSF Aβ_42_ analysis, and Stage 2: Neurodegeneration by PET and CSF tau (Jack et al., 2010). The preclinical phase can also be separated into “pre-symptomatic” and “asymptomatic at risk” suggested by IGW−2014 (Dubois et al., 2014). The pre-symptomatic preclinical AD refers to individuals with familial AD who will develop AD in the future. Individuals with pre-symptomatic preclinical AD show no clinical symptoms but have at least one mutation in the familial AD genes (APP, PSEN1, PSEN2). Asymptomatic at risk refers to preclinical AD in individuals without clinical symptoms, but positive for AD biomarkers (decreased level of Aβ_42_, increased the level of tau p-tau in CSF) or positive in Aβ-PET (Dubois et al., 2014). According to the NIA-AA (2011) progressive preclinical AD pathological trajectory can be divided into three distinguishable stages: in the first stage, there is evidence of abnormality in Aβ, and individuals in this stage would be positive for Aβ, no dementia or neurodegeneration. The second stage consists of positive for Aβ, plus higher CSF tau (neurodegeneration). In the third stage, individuals begin exhibiting evidence of memory problems along with abnormalities in CSF biomarkers and neuroimaging, but all evidence of memory problems is less than MCI cases (Sperling et al., 2011).

## Critical evaluation of IGW and NIA-AA preclinical Alzheimer's disease criteria

AD pathology is a continuum process spanning many years of underlying changes in brain morphology due to preclinical AD stage to a clinical AD phase. Hippocampus volume loss, temporoparietal hypometabolism, and neocortical Aβ deposition are the first to be affected in brain areas due to preclinical AD pathology. Metabolic brain networks of these areas are affected by age as well as by preclinical AD. White matter brain network is the primary area that is also affected in preclinical AD. On the cellular level, the synaptic and the axonal degenerations start to occur, but such degenerations would not affect the overall memory at the preclinical AD stage. The critical point is how and when the *in-vivo* AD-related pathology that determines preclinical AD can be measured by AD-biomarkers. The revised IWG definition of a preclinical AD: no clinical sign of AD but has at least one positive pathological AD biomarker (Dubois et al., 2016). In a critical viewpoint, it will be very difficult to distinguish preclinical AD by only one AD-biomarker. On the other hand, the NIA-AA definition of preclinical AD is more generalized (not specific): evidence of amyloidosis and neurodegeneration (Sperling et al., 2011). Here also we need the more specific definition of the role and applicability of AD *in-vivo* pathological biomarkers. The revised IWG definition of preclinical AD is too simplified to find the right condition to diagnose preclinical AD. Therefore, neither of them are perfect for determining *in-vivo* preclinical AD by AD pathological biomarkers. Both IGW and NIA-AA definition of preclinical AD considered amyloidosis ahead of tauopathy. There is evidence that neurodegeneration due to high tau or tauopathy started before amyloidosis (Jack and Holtzman, 2013; Jack et al., 2013). The phenomenon of mixed pathologies (AD with non-AD dementia) underlying the preclinical AD stage of neurodegeneration has been completely ignored by both (IGW and NIA-AA) the definitions of preclinical AD.

## Hypothetical model of preclinical Alzheimer's disease and progression of Alzheimer's disease

**Figure 1:**
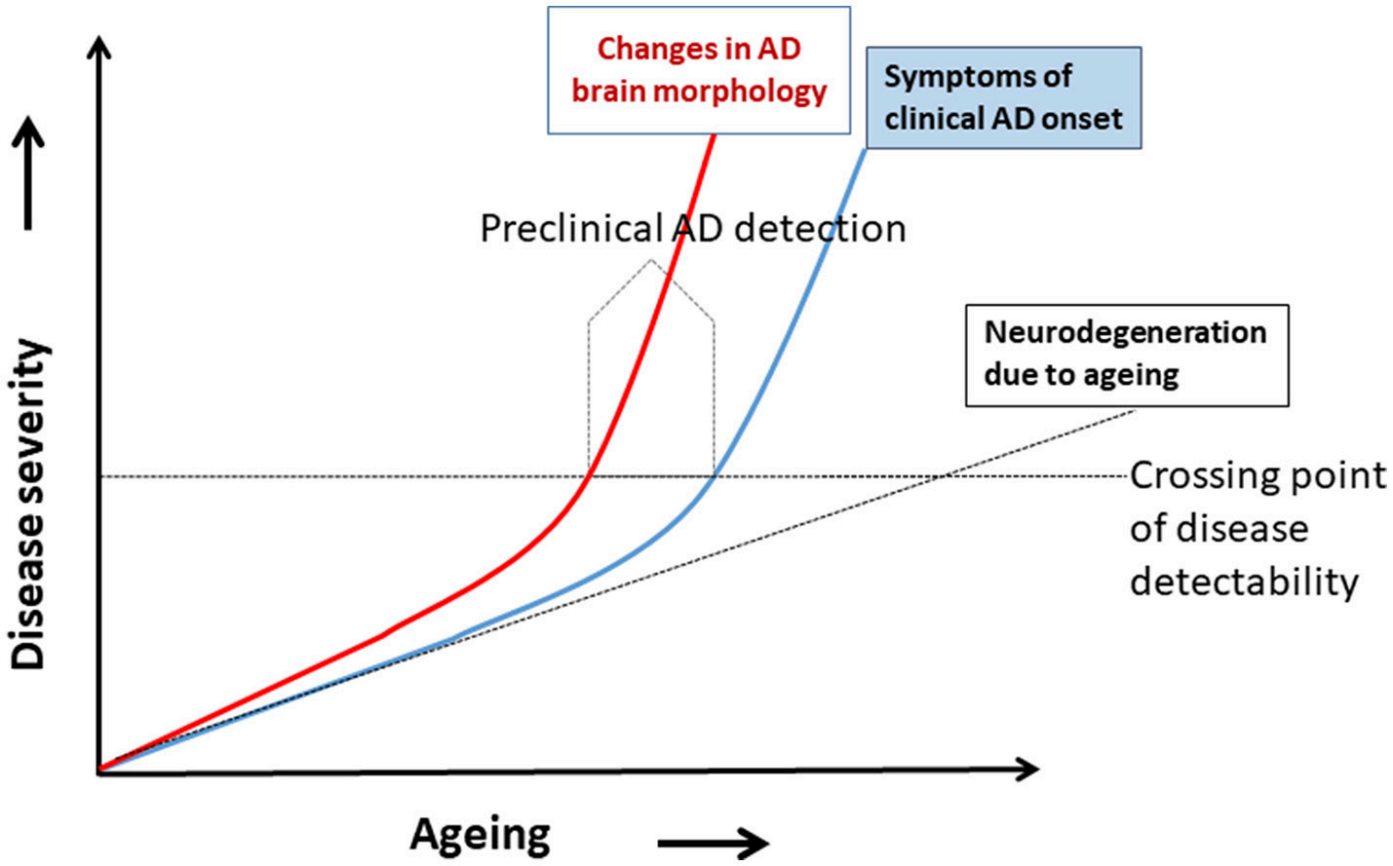
A hypothetical model for detecting preclinical Alzheimer's disease (AD). Disease severity increases with aging, the major risk factor for an AD. Two disease trajectories represent brain morphology changes (red) and clinical AD symptoms (blue). Neurodegeneration due to aging shown by the broken black line. The model shows the hypothetical deviation of the changes in brain morphology trajectory and symptoms trajectory, and where they cross a horizontal line of disease detectability. The preclinical AD can be detected before the onset of AD symptoms (modified from Khan, 2016).

A hypothetical model of AD progression in relation to aging and disease severity is shown in Figure 1. The disease severity increases as age increases, because age is the most important risk factor for AD dementia. Two trajectories of AD progression are shown, one based on changes in brain morphology (red) and the other is the onset of clinical symptoms of neurodegeneration (blue). Neurodegeneration due to normal aging is shown as a broken black line. The model shows deviations of the brain morphology trajectory and the clinical symptoms trajectory as they cross a hypothetical line of disease detectability. The trajectory of neurodegeneration due to normal aging crosses this line much later in life. Note that the changes in brain morphology increased much earlier than clinical symptoms; this indicates that the pathological changes and abnormalities in brain cell signaling (neuronal network function) occur earlier in disease progression than symptoms, such that CSF biomarkers, for example, would be detectable even in the absence of symptoms. Preclinical AD could thus be detected before the onset of AD symptoms (Figure 1). Preclinical diagnosis of AD is possible when brain morphology due to AD pathology started to change (box “Preclinical AD detection” in Figure 1), (subtle changes in brain white matters, minute changes in brain metabolites, and abnormalities in the neuronal network by functional MRI) and biomarkers of brain morphology changes can detect such signals. It is at this point in disease progression that advanced non-invasive neuroimaging of morphologic biomarkers with modalities, such as rs-fMRI, DTI MRI, ASL MRI, and PET, have enormous potential for diagnosis of preclinical AD.

The abnormalities in the brain due to the onset of AD pathology start 10–20 years before symptoms of cognitive deficiencies appear in genetically susceptible cases (Morris, 2005; Reiman et al., 2012). In one study, brain MRI was used to image brain atrophy in individuals classified as asymptomatic AD mutation carriers (presymptomatic preclinical). A recent study found that brain atrophy could be detected 1–8 years before the clinical symptoms of familial AD appeared (Kinnunen et al., 2017). A similar approach to detecting preclinical AD based on MRI-measured brain atrophy over time is not as simple for sporadic AD, however. More research is necessary to validate the preclinical AD category of “asymptomatic at risk.” Brain amyloidosis (higher Aβ deposition) in a cognitively normal individual has a higher likelihood of progression from preclinical stages to symptomatic stages of AD.

## Applicability of cerebrospinal fluid (CSF) biomarkers in preclinical Alzheimer's disease diagnosis

Widely researched CSF AD biomarkers are low Aβ_42_, high tau, and high p-tau (phosphorylated tau) levels compared to age-matched non-demented controls. Three CSF biomarkers represent three different aspects of AD brain pathology, respectively. Low levels of Aβ_42_ reflects higher amyloid plaques (amylolysis), high levels of tau represents neurodegeneration, and high p-tau level correlates with high levels of neurofibrillary tangles (NFTs) in the AD brain. As a single test CSF biomarkers are not accurate to detect conversion of MCI to AD; however, negative CSF results in MCI cases can accurately predict conversion of non-Alzheimer's disease (Ritchie et al., 2017). Moreover, an evaluation of CSF biomarkers found them to have the ability to predict memory decline in individuals with aging (Fagan et al., 2007; Gustafson et al., 2007; Stomrud et al., 2007; Jansen et al., 2015). Therefore, CSF biomarkers can be used as part of a preclinical biomarker panel that can predict developing AD in the future.

CSF biomarker patterns of preclinical AD were found to be different in middle aged asymptomatic cases in a recent longitudinal study (Sutphen et al., 2015). Besides established AD CSF, (Aβ_42_, tau, and p-tau) there are other CSF biomarkers that are more close to the neurodegeneration in preclinical AD stage. Such recently studied CSF biomarkers are neurofilament light chain, neurogranin, inflammatory markers, and tau fragments. Levels of CSF neurogranin are high in AD and progressive AD cases (Kester et al., 2015). Higher levels of neurogranin represent synaptic dysfunction and neurodegeneration. Neurogranin level was correlated with tau but not Aβ, which indicates it can be a measure of neurodegeneration, not levels of Aβ deposition. The levels of neurofilament light chain concentration were also high in AD progression (Zetterberg et al., 2016). Neurofilament light chain is a measure of axonal degeneration due to underlying preclinical AD. CSF inflammatory markers such as IL-15, MCP-1, VEGFR-1, sICAM1, sVCAM-1, and VEGF-D were found to correlate with the levels of tau and p-tau (Popp et al., 2017). More research and validation is needed for these newly incorporated CSF biomarkers before considering them definitive preclinical AD biomarkers. Longitudinal follow-up of CSF biomarkers in individuals in risk cohort will be more effective than cross-sectional cohort for determining preclinical AD progression.

## Advanced MRI and PET-based neuroimaging for the diagnosis of preclinical Alzheimer's disease

Non-invasive imaging of the brain is a promising tool for the early detection of AD. The most widely researched neuroimaging biomarkers of AD are summarized in Table 1. Advanced neuroimaging techniques are most promising in detecting the disease at its earliest stage for initiating therapeutic intervention and finding individuals at risk of AD. In fact, hippocampus volume is one of the first brain areas affected due to preclinical AD pathology. Advanced MRI-based neuroimaging techniques and protocols have been introduced to detect more subtle changes in brain tissues at the microscopic level (Table 2). Tissues and cell damage that precede neurodegeneration include loss of synapses, loss of axonal integrity, demyelination, loss of microtubule assembly, and minute changes in levels of brain metabolites. Resting-state functional MRI (rs-fMRI), DTI, and ASL MRI have been developed to detect these preclinical AD biomarkers and are described here. Longitudinal follow-up of imaging biomarkers in individuals in an at risk cohort will be more effective than a cross-sectional cohort for determining preclinical AD progression.

**Table 1:** Neuroimaging biomarkers in Alzheimer's disease.

**Neuroimaging biomarker**	**Biomarker readout associated with AD**
MRI^*^	Atrophy
fMRI	Disrupted neural network
^11^C-PIB PET^*^	Increased amyloid plaque binding to PIB
^18^F-Aβ-binding compound-PET^**^	Higher amount of amyloid in brain
^18^FDG-PET^*^	Low brain metabolism measured by decreased glucose uptake

*Aβ, amyloid beta; AD, Alzheimer's disease; FDG, fluoro-2-deoxy-D-glucose; fMRI, functional MRI; MRI, magnetic resonance imaging; PET, positron emission tomography; PIB, Pittsburgh compound B*.

* *Included in the NIA-2011 and IWG−2014 criteria to support diagnosis of AD for research purposes*.

** *Approved by FDA and EMA*.

**Table 2:** Preclinical Alzheimer's disease neuroimaging biomarkers.

**Modality**	**Parameter**	**Values in preclinical AD**	**Notes**	**References**
rs-fMRI	Paramagnetic property of oxy-hemoglobin/deoxy-hemoglobin in blood flow	Low-frequency spontaneous fluctuation of BOLD signal Reduced functional connectivity	rs-fMRI can detect abnormalities before brain volume loss No regulatory guidelines by FDA or EMA	Machulda et al., 2011; Sheline and Raichle, 2013; Buckley et al., 2017
DTI with sMRI	Diffusion of water molecules in brain	Higher overall mean diffusivity of water molecules Lower fractional anisotropy	Region specificity Detectable abnormality in WM networks in preclinical AD No regulatory guidelines by FDA or EMA	Sexton et al., 2011; Racine et al., 2014; Fischer et al., 2015; Nedelska et al., 2015
ASL/sMRI	CBF traced by magnetically labeled water molecules	Decreased blood flow in brain	Consistent with vascular hypothesis of AD Could be useful biomarker for tracking disease severity and progression No radiotracer or contrast reagents No regulatory guidelines by FDA or EMA	Alsop et al., 2010; Wang et al., 2013; Wierenga et al., 2014; Hays et al., 2016
Aβ-PET	Radiotracer binding to amyloid beta in specific brain regions	Presence of Aβ detected with ^11^C-PIB or ^18^F-labeled amyloid-binding agents	Amyloid accumulation occurs before brain atrophy and is hypothesized to be the first pathological event in AD It is not correlated with AD clinical severity and neurodegeneration Can be used for differential diagnosis per FDA or EMA	Jack et al., 2009; Jagust et al., 2009; Scheinin et al., 2009; Okamura et al., 2014
^18^FDG-PET	Glucose uptake in different brain regions	Glucose uptake using ^18^FDG	Useful for differential diagnosis of AD and FTD Brain region-specific differentiation capability Can be used for differential diagnosis per FDA or EMA	Mosconi et al., 2006; Ewers et al., 2013, 2014
Tau-^18^F-T807-PET	Region specific distribution of tau deposition	^18^F-T807	Progression of AD related to neurodegeneration due to tau deposition	Johnson et al., 2016

*Aβ, amyloid beta; AD, Alzheimer's disease; ASL MRI, arterial spin-labeled magnetic resonance imaging; BOLD, blood oxygen level-dependent; CBF, cerebral blood flow; DTI, diffusion tensor imaging; EMA, European Medical Agency; FDA, Food and Drug Administration; FTD, frontotemporal dementia; FDG, fluoro-2-deoxy-D-glucose; fMRI, functional MRI; MRI, magnetic resonance imaging; ^18^F-T807-PET, Fluorinated tau PET ligand; PET, positron emission tomography; PIB, Pittsburgh Compound B; rs-fMRI, resting state functional MRI; sMRI, structural MRI; WM, white matter*.

### Resting-state functional MRI (rs-fMRI)

Resting-state functional MRI was first developed by Biswal et al. (1995) to detect low-frequency fluctuations in the resting brain. In principle, it measures changes in paramagnetic properties of oxyhemoglobin/deoxy-hemoglobin in blood flowing through different brain regions influenced by changes in neuronal network activity. In the resting brain, the neuronal network is linearly dependent on spontaneous low-frequency fluctuations of the blood oxygen-dependent (BOLD) signals detected by fMRI in different brain areas. In a task-free state, functional connectivity analysis can detect subtle changes in brain network differences between individuals with the early-stage disease and healthy controls. The neuronal network and synaptic activities are beginning to change in preclinical AD, before the symptomatic manifestation of AD. Thus, rs-fMRI enables functional connectivity analysis to detect subtle brain network abnormalities in the very beginning of AD pathology in the brain. Recently, lower functional connectivity measured by rs-fMRI has also been demonstrated to be an indicator of pre-MCI and pre-dementia due to AD in longitudinal studies of individuals with preclinical AD (Buckley et al., 2017) (Table 2).

### Diffusion tensor imaging (DTI) with sMRI

DTI, sometimes called diffusion-weighted imaging (DWI), was developed in the last decade of the twentieth century (Moseley et al., 1990; Beaulieu and Allen, 1994; Pierpaoli and Basser, 1996; Pierpaoli et al., 1996). In principle, it measures the probability distribution of diffusion of water molecules in the brain in terms of the diffusion tensor. In the ideal case, if there are no hindrances, the probability distribution of diffusion of water molecules should be isotropic. A brain has nerve fibers and tightly associated axonal bundles; therefore, the distribution of water molecules should be highly anisotropic in non-demented normal brains. The phenomenon of white matter loss starts at the preclinical AD stage. This loss of white matter decreases the anisotropic nature of the diffusion of the water molecules. DTI calculates two measurable quantities from the anisotropic nature of diffusion of water molecules by sMRI in the region of interest in the brain. Those two quantities are fractional anisotropy (FA) and mean diffusion coefficient (MD). Increased MD and decreased FA values indicate loss of white matter in AD and MCI brains indicating that MD and FA may be potential neuroimaging biomarkers of early-stage AD. Several studies have shown that DTI has the ability to distinguish between AD, MCI, and age-matched control case (Chua et al., 2009; a meta-analysis of 41 DTI studies by Sexton et al., 2011) (Table 2).

Expanding on this work, DTI technology combined with novel mathematical tools has been used recently to identify preclinical AD cases. New evidence suggests that white matter alterations begin in preclinical AD and can be measured by DTI (Racine et al., 2014; Kantarci et al., 2017). White matter primarily consists of axon and myelin sheets that is altered by AD pathology. Axonal degeneration and deformation of myelin sheets are early events that occur before the wide-spread neuronal loss in the AD brain. Among the neuroimaging techniques, DTI is the best suited to assess degeneration of myelinated nerve fibers in the brain. By applying tractography and graph theory in DTI, a study reported alterations within the entire white matter network in preclinical AD, even before structural markers of significant neurodegeneration, such as atrophy by MRI and reduced cortical glucose utilization by ^18^FDG-PET, were detected (Fischer et al., 2015). Moreover, the alteration of DTI parameters (FA and MD) were correlated with common risk factors of sporadic AD (Adluru et al., 2014).

### Arterial spin labeling (ASL) MRI

ASL/MRI imaging of AD is consistent with the hypothesis of vascular abnormality of the AD. ASL was first developed in 1992 for imaging the rat brain using magnetically labeled blood water (arterial spin labeling) followed by MRI (Detre et al., 1992). The principle of ASL is based on imaging magnetically labeled blood water in brain tissues of a region of interest by applying a 180° radio-frequency pulse (Detre et al., 2009, 2012). In the next step, the local changes of magnetization in brain tissue by blood flow with magnetically labeled water are measured by scanning with normal MRI sequence scanning mode. It has the ability to identify vascular factors in neurodegenerative diseases such as AD and vascular dementias (Detre et al., 2012). Cerebral blood flow (CBF) is a possible biomarker of AD that can be measured by ASL MRI. Typical CBF images by ASL MRI measure reduced CBF in AD, and can even differentiate between MCI, and age-matched control groups (Wang et al., 2013). Reduced CBF values measured by ASL MRI were found to be region specific AD patients compared to age-matched control cases. Reduced CBF occurs in the lateral prefrontal cortex, posterior cingulate, precuneus, and inferior parietal areas of AD brains (Alsop et al., 2000, 2010; Johnson et al., 2005; Dai et al., 2009) (Table 2). CBF is easy to follow over time and imaging a biomarker can be useful to follow the disease's condition and prognosis. It has been hypothesized that abnormality in CBF occurs much earlier than cognitive deficits appear in AD, possibly earlier than wide-spread brain atrophy or plaque/tangle formation. Recent studies showed that ASL MRI can be extended to detect preclinical AD, at least for research purposes (Wierenga et al., 2014; Hays et al., 2016).

## AD brain imaging by positron emission tomography (PET)

### Amyloid imaging by PET as AD biomarker

Aβ deposition can be found in the neocortical area of the brain, one of the first areas affected due to preclinical AD pathology. Imaging of amyloid as a biomarker of AD is based on the popular yet controversial “amyloid cascade hypothesis” of AD (Hardy and Allsop, 1991; Hardy and Higgins, 1992). According to this oversimplified hypothesis, toxic deposition of amyloid in the AD brain causes synaptic loss and neuronal apoptosis; thus, measuring Aβ aggregates in antemortem AD brain imaging by PET would give the right AD diagnosis (Table 2). This hypothesis also supports the idea that amyloid plaque deposition is the primary pathophysiologic change that occurs in preclinical AD. Since fibrillary tau deposition is common in another neurodegenerative disease (Tauopathy, Frontotemporal dementia, Corticobasal degeneration), amyloid imaging by tau would have higher specificity. Klunk et al. (2004) were first to develop ^11^C-PIB (2-(4′-[11C] methylaminophenyl)-6-hydroxybenzothiazole or Pittsburgh compound B) as an Aβ PET imaging agents (Klunk et al., 2004). The ^11^C-PIB-PET imaging biomarker has a limitation in terms of sensitivity specificity. A study found ^11^C-PIB-PET positive in about 20–30% of cases of cognitively normal individuals (Pike et al., 2007). On the other hand, a report demonstrated that ~16% of patients with probable AD were PIB PET negative (Shimada et al., 2011). As Aβ plaque is not associated with dementia in AD, the earliest event in preclinical AD, synaptic loss, would not be expected to correlate with ^11^C-PIB-PET alone. A longitudinal study of PIB-PET found no correlation of PIB uptake depending their dementia status in age-matched control, MCI, and AD individuals (Jack et al., 2009). Therefore, ^11^C-PIB-PET may provide evidence of amyloid deposit in the brain but may not be useful for diagnosing preclinical AD alone. Another Aβ PET imaging compound, ^18^F-florbetapir demonstrated greater specificity than CSF Aβ_42_ (Mattsson et al., 2014). The half-life of positron-emitting ^11^C-PIB (20.33 min) is much lower than that of ^18^F (109.77 min). While ^18^F compounds provide a shorter window for conducting an imaging study, this also means that the ^18^F radiotracer must be made in-house using a cyclotron, or within a range consistent with the 20-min half-life. Other well studied Aβ radiotracers are ^11^C-BF227 and ^18^F-NAV4694. A recent study found that Aβ-PET is suitable for defining preclinical AD along with CSF biomarkers (Dubois et al., 2016). Moreover ante mortem ^11^C-PIB-PET scanning results were correlated with the Thal amyloid deposition stages (Murray et al., 2015). Amyloid plaque score in terms of Thal stage has been incorporated in NIA-AA (2011) AD neuropathological criteria (Hyman et al., 2012).

### Biomarker of glucose metabolism in AD by PET

Dysfunctional brain glucose metabolism is another hypothesis of AD pathogenesis. Temporoparietal hypometabolism is one of the brain areas first affected due to preclinical AD pathology. [^18^F]-fluoro-2-deoxy-D-glucose (^18^FDG) has been extensively used as the PET agent for region-specific brain imaging. ^18^FDG-PET imaging outcomes from AD patients were appropriately correlated with the mini-mental score examination (MMSE) (Jagust et al., 2009). In fact, among the PET imaging techniques, ^18^FDG-PET is the most widely researched imaging biomarker for AD. ^18^FDG-PET showed consistent low signal in the parietal-temporal area and posterior cingulate cortex. In severe AD, the frontal cortex showed lower signal; however, other brain areas unaffected by AD pathologies such as the cerebellum, striatum basal ganglia and the visual and sensory cortex remained unchanged. A differential low glucose metabolism detected at different brain region by ^18^FDG-PET can be used for differential AD and non-AD dementia diagnosis (Silverman et al., 2001; Mosconi et al., 2008). ^18^FDG-PET can be used for differential AD diagnosis vs. other non-AD dementia suggested by important regulatory authorities, such as FDA and EMA. In addition to that, International panels for AD diagnostic criteria have included ^18^FDG-PET as one of the AD biomarkers (Sperling et al., 2011; Dubois et al., 2014). The Centers for Medicare and Medicaid Services (CMS) have allowed the use of ^18^FDG-PET to establish the diagnosis of dementia due to AD and frontotemporal dementia (FTD). ^18^FDG-PET also has potential to predict preclinical AD pathology (Ito et al., 2015).

### Biomarkers of tau imaging in AD by PET

The hyperphosphorylated paired helical filament (PHF) that forms NFTs quantified by Braak stages are better correlated with AD severity and neuronal atrophy than amyloid plaques (Bierer et al., 1995; Nelson et al., 2007). Both tau and phosphorylated tau (p-tau) are increased in AD pathology considered to be the measure of neuronal injury. Some studies even showed that neuronal injuries due to tau and p-tau are earlier than abnormalities in amyloidosis (Jack and Holtzman, 2013; Jack et al., 2013). CSF tau is not the same as deposition of NFTs in the AD brain. Moreover, the dynamic ranges of tau and p-tau in CSF AD biomarkers are lower than Aβ_42_. Therefore, tau imaging by PET as AD preclinical should have a higher implication.

Tau-PET imaging for AD was initiated with^18^FDDNP (a radiofluorinated derivative of the 2-(1-[6-(dimethylamino)-2-naphthyl]ethylidene) malononitrile) showing higher binding in AD and MCI cases (Small et al., 2006). The first human trial of tau radiotracer ^18^F-T807 by Siemens Molecular Imaging Biomarker Research (Culver City, CA) found higher tau levels in brain areas rich in PHF and very low in white matter (Chien et al., 2013). There are several other new tau-PET tracers that have been developed based on quinolone derivatives (^18^F-THK-523, ^18^F-THK-5105, and ^18^F-THK-5117) (Fodero-Tavoletti et al., 2011; Harada et al., 2013; Okamura et al., 2014). Longitudinal tau-PET cohorts in patients with high-risk preclinical AD provided special distribution of deposition of tau that can allow to staging *in-vivo* neurodegeneration according to tau levels in preclinical AD (Johnson et al., 2016). Special distribution of tau deposition by tau-PET would serve a very important role in determining preclinical AD cases. For example, tau-PET ligand uptake in the neocortex and increase amyloidosis with time in longitudinal studies can find underlining preclinical AD pathology. Tau-PET uptake in the medial temporal lobe can be due to non-AD pathology preclinical FTD (frontotemporal dementia), CBD (corticobasal degeneration), PSP (progressive supranuclear palsy), or age-related tauopathy (Crary et al., 2014). Tau-PET binding in medial temporal lobe is not useful for preclinical AD diagnosis since it cannot differentiate control from MCI cases. Moreover, Tau-PET binding occurs in media temporal lobe even in asymptomatic elderly cases up to Braak stage 2.

## Applicability of brain neuroimaging modalities for detection of biomarkers in preclinical Alzheimer's disease

A comparison of the different neuroimaging modalities used to detect preclinical AD biomarkers is shown in Table 3. ^18^FDG-PET imaging outcome determines region-specific subtle metabolic changes in the brain. According to metabolic dysfunction hypothesis AD, pathologic changes first occur in the metabolic pathway of a preclinical AD brain. While ^18^FDG-PET correlates strongly with AD and AD progression, it has the potential to distinguish AD vs. other non-AD dementia, and it strongly correlates with MMSE. Therefore, ^18^FDG-PET has the capability to distinguish preclinical AD. There will be some disadvantages using ^18^FDG-PET for the diagnosis of preclinical AD. The ^18^FDG-PET signal can be affected by inflammation, local ischemia, and the behavior state of the subject (Duara et al., 2010; Shipley et al., 2013). The diagnostic criteria for the preclinical AD by IWG-AA (2015) does not list either MRI or ^18^FDG-PET as suitable modalities for defining preclinical AD (Dubois et al., 2016). However, IWG-AA recommends^18^FDG-PET for tracking progress in clinical AD in individuals with asymptomatic preclinical AD (Dubois et al., 2016).

**Table 3:** Comparison of preclinical Alzheimer's disease neuroimaging modalities.

**Neuroimaging modalities**	**Differences**	**References**
Tau-^18^F-T807-PET vs. ^13^C-PIB-PET	Region specific distribution of tau deposition	Johnson et al., 2016
Tau-^18^F-THK5317-PET vs. ^18^FDG-PET	Progression of AD is better tracked by ^18^FDG-PET	Chiotis et al., 2017
^18^FDG-PET vs. DTI with sMRI	Whole-brain white matter network properties in preclinical AD can be detected by DTI with sMRI before structural markers of significant neurodegeneration such as atrophy (by MRI) or reduced cortical glucose utilization (by ^18^FDG-PET)	Fischer et al., 2015
^18^FDG-PET vs. ASL\MRI	ASL MRI is not invasive ^18^FDG-PET is more expensive than ASL/MRI ASL/MRI operation is simple	Wolk and Detre, 2012

*Aβ, amyloid beta; AD, Alzheimer's disease; ASL MRI, arterial spin-labeled magnetic resonance imaging; DTI, diffusion tensor imaging; FDG, fluoro-2-deoxy-D-glucose; fMRI, functional MRI; ^18^F-T807-PET, Fluorinated tau PET ligand; Tau-^18^F-THK5317, Fluorinated tau PET ligand; MRI, magnetic resonance imaging; PET, positron emission tomography; PIB, Pittsburgh compound B*.

rs-fMRI can detect abnormalities before brain volume loss (atrophy as determined by sMRI). It can be used to detect reduced functional connectivity in the preclinical stage of AD. However, there are no regulatory guidelines by FDA or EMA. DTI/sMRI has the potential to detect abnormalities in white matter networks in preclinical AD. In addition, it has the capability to detect region specificity to detect a specific region affected by preclinical AD. ASL/MRI can detect the abnormality in CBF in the preclinical AD. While Aβ-PET imaging can only be a signature of an amount of Aβ levels in the preclinical AD brain, it may not be used to detect the level of neurodegeneration (Table 3).

## Risk factors in preclinical Alzheimer's disease

Preclinical AD can be classified in terms of degree of the risk factor of developing preclinical AD (except age). Such risk factors are positive amyloidosis, positive neurodegeneration, abnormalities in synaptic function by fMRI, and positive genetic AD risk factors. Individuals with high-risk factors should be included in a longitudinal follow-up in a cohort to find the relationship between risk factor and development of preclinical AD. A longitudinal cohort study showed CSF AD biomarkers (Aβ_42_ and tau) and Aβ-PET can distinguish preclinical AD as high risk and low-risk categories (Vos et al., 2013). In future patients in a high-risk AD category and preclinical categories can be advised to change their lifestyle and food habits to delay the disease. Longitudinal changes with different risk factor in preclinical AD follow-up until cognitively impaired will allow for estimating positive and negative predictive values of the use of AD biomarkers in an at-risk population.

## Need for diagnostic guidelines for preclinical AD

Ample evidence shows that AD has long prodromal stages (preclinical AD → MCI due to AD pathology → AD-dementia) before the real clinical manifestation of dementia. The earlier treatment of AD begins in the disease course, the more effective it is at slowing the progression of the disease. Therefore, the detection of preclinical AD in asymptomatic individuals has become a major AD research focus. A simple definition of preclinical AD by the International panel (IWG-2014): No clinical symptoms of AD but positive AD biomarker values (in CSF: decreased Aβ_42_, increased tau and/or p-tau in CSF; or in brain imaging: increased fibrillary amyloid on PET; Dubois et al., 2014). This simplistic definition needs more extensive diagnostic guidelines for preclinical AD. Deposition of amyloid plaques in the brain begins decades before clinical manifestation of AD, and the NIA-AA (2011) defines preclinical AD on the basis of pathological changes in the brain that occur before a demonstration of cognitive deficits. Later, IWG and NIA-AA (2015) simplified the definition of preclinical AD as the period of time between the first evidence of neuropathological lesions in the brain to the date of first clinical symptoms of AD. Now the challenge is to find validated biomarkers capable of detecting the first evidence of neuropathological lesions in the brain. Extensive research of AD biomarkers over the last couple decades has shown that preclinical AD is more likely to be diagnosed using multi-modal criteria. There is a need for evidence-based guidance on how to combine validated tests and imaging modalities to diagnose AD before the widespread synaptic loss and irreversible neuronal damage occur.

A combination of early Aβ-PET, ^18^FDG-PET, and DTI MRI and/or fMRI to detect neurodegeneration, supported by genetic tests of the mutated APP, PSEN1, PSEN2, and APOE4, may be appropriate for preclinical AD diagnosis. Positive Aβ-PET, low ^18^FDG-PET, and the presence of subtle neurodegeneration on MRI has been detected in familial AD prior to clinical symptoms. In sporadic AD, it would be reasonable to categories individuals with APOE4, positive Aβ-PET, low ^18^FDG-PET, and subtle neurodegeneration by MRI as having a preclinical AD. Diagnosis of preclinical AD in an individual without genetic markers but with positive Aβ-PET, low ^18^FDG-PET, and subtle evidence of neurodegeneration on MRI would have to wait until subtle evidence of cognitive decline appears that is not yet equivalent to the level indicative of MCI.

## A possible algorithm for comprehensive preclinical Alzheimer's disease diagnosis

**Figure 2:**
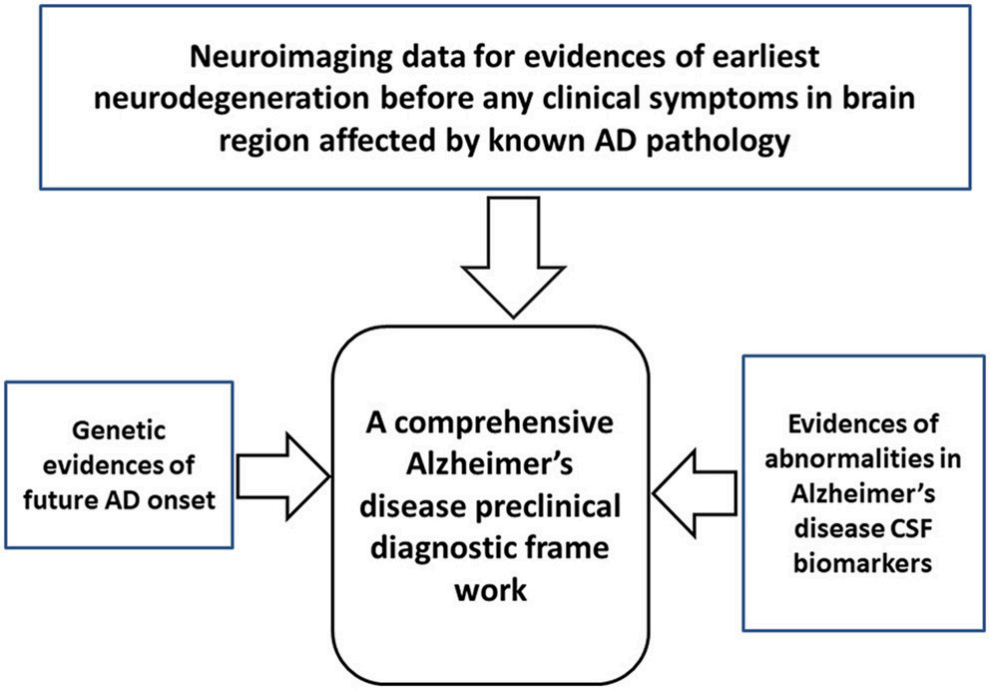
A simple framework for comprehensive diagnosis preclinical Alzheimer's disease (AD). The framework includes three diagnostic modalities: neuroimaging to detect the earliest evidence of neurodegeneration in brain areas susceptible to AD pathology, genetic markers associated with an AD, and biomarker testing to detect abnormalities associated with an AD.

How and at what point in the lifespan can we begin to detect clear-cut signs of the ongoing neurodegenerative process of AD pathology that is distinct from normal aging? A multidimensional “panel” of preclinical AD biomarkers presents the best chance for a diagnosis and prediction of progression to AD dementia. A combination of three sets of evidence is recommended: (1) neuroimaging to detect early evidence of neurodegeneration in brain areas susceptible to AD pathology; (2) the genetic risk markers that predict AD onset; and (3) evidence of abnormalities in AD biomarkers (e.g., CSF Aβ_42_, tau, and p-tau) (Figure 2). Major exclusion criteria would not be useful for preclinical AD diagnosis. Only deposition of tau at the medial temporal lobe by tau-PET can be used as an exclusion of preclinical AD from other tau related preclinical non-AD dementia (preclinical FTD, CBD or age-related tauopathy). Preclinical AD diagnostic algorithm has been proposed based on this combinatorial approach of neuroimaging, genetic testing, and CSF biomarker tests (Table 4). Incorporation of biomarkers and genetic information into the preclinical AD diagnostic scheme may also permit prediction of the *in vivo* physiological changes occurring in the brain before a clinical AD diagnosis.

**Table 4:** Algorithm for preclinical Alzheimer's disease diagnosis.

**Approach**	**Preclinical AD biomarkers**	**Preclinical AD diagnosis**
Pre-symptomatic genetic risk factors of familial AD	Familial AD genes: APP, PSEN1, PSEN2	Definite preclinical AD
Combination of sporadic AD risk factor genes (APOE4), CSF biomarkers, and neuroimaging biomarkers	APOE4, TOMM40: Yes CSF: low Aβ_42_, high tau and p-tau Neuroimaging: high Aβ by PET, neurodegeneration by low ^18^FDG-PET	Highly probable preclinical AD
Combination of sporadic AD risk factor genes (APOE4), CSF biomarkers, and neuroimaging biomarkers	APOE4: No CSF: low Aβ_42_, high tau and p-tau Neuroimaging: high Aβ by PET, neurodegeneration by low ^18^FDG-PET	Probable preclinical AD
Combination of sporadic AD risk factor genes from Alzgen data base, CSF biomarkers, and neuroimaging biomarkers	Susceptible gene: Alzgene database CSF: low Aβ_42_, high tau and p-tau Neuroimaging: high Aβ by PET, neurodegeneration by low ^18^FDG-PET, deposition of tau in cortical region by ^18^F-T807-PET	Probable preclinical AD
Combination of sporadic AD risk factor genes (APOE4), CSF biomarkers, and neuroimaging biomarkers	APOE4: No CSF: low Aβ_42_, high tau and p-tau Neuroimaging: high Aβ by PET, no neurodegeneration by ^18^FDG-PET, deposition of tau in cortical region by 18F-T807-PET	Suspected preclinical AD
Combination of sporadic AD risk factor genes (APOE4), CSF biomarkers, and neuroimaging biomarkers	APOE4: No CSF: no Aβ_42_, high tau and p-tau Neuroimaging: low or no Aβ by PET, neurodegeneration by low ^18^FDG-PET	Suspected preclinical non-AD pathophysiology

*Alzgene (http://www.alzgene.org/); Aβ, amyloid beta; APOE, apolipoprotein; CSF, cerebrospinal fluid; FDG, fluoro-2-deoxy-D-glucose; ^18^F-T807-PET, Fluorinated tau PET ligand; PET, positron emission tomography; TOMM40, translocate of outer mitochondrial membrane 40*.

Validation of this framework will require accurate identification of an asymptomatic cohort at risk of AD. Longitudinal follow-up of different risk factors in preclinical AD will allow estimating accuracy, sensitivity, specificity, positive, and negative predictive values of the use of a particular AD biomarker at-risk population. The applicability of this diagnostic algorithm for screening of preclinical case needs extensive validation. Predicting positive and negative predictive values and false positives will depend on how preclinical AD cases will be selected. Standardization of operating procedure, thresholds, and cutoff values of CSF and neuroimaging biomarkers are needed to minimize between-lab and between-batch variability. Despite these challenges, the use of biomarkers holds great promise for the detection of the preclinical AD and the initiation of therapy at earlier stages to slow the progression of the disease. CSF sample collection is highly invasive and neuroimaging biomarkers are expensive; therefore, the ultimate goal for this research area is to find peripheral preclinical biomarkers for AD.

## Issues concerning preclinical AD biomarkers

There are several necessary issues of preclinical AD biomarkers to be addressed before application. Most important issues are diagnostic accuracy, patient selection in clinical trial, universal standardization of diagnostic protocols, cost of diagnosis, the complexity of patient selection in clinical trials, and ethical challenges.

### Diagnostic accuracy and patient selection

Improved diagnostic accuracy (sensitivity, specificity, positive predictive value, negative predictive value) and universally accepted unified standard operating procedures (SOP; for sample collection, cut-off values, analysis) are urgently required. The diagnostic sensitivity (SN), specificity (SP), positive likely-hood ratio (LR+), and negative likely-hood ratio (LR–) of all three core CSF biomarkers (Aβ_42_: SN = 79%, SP = 63%, LR+ = 2, and LR– = 0.3; tau: SN = 76%, SP = 58%, LR+ = 2, and LR– = 0.4; p-tau: SN = 78%, SP = 56%, LR+ = 2, and LR– = 0.4; combination of three: Aβ_42_: SN = 84%, SP = 63%, LR+ = 2, and LR– = 0.3) for MCI conversion to AD were found to be moderate by a systemic meta-analysis (Ferreira et al., 2014). The diagnostic sensitivity, specificity, positive likely-hood ratio, and negative likely-hood ratio of ^18^F-labeled Aβ-PET imaging biomarkers (Florbetapir: SN = 89.6%, SP = 87.2%, LR+ = 7.9 and LR– = 0.108; Florbetaben: SN = 89.3%, SP = 87.6%, LR+ = 6.06 and LR– = 0.141) for distinguishing AD with non-demented control were found to be considerably higher by a systemic meta-analysis (Yeo et al., 2015). The progression of AD in non-demented elderly individuals was predicted with considerable accuracy (SN = 82% and SP = 93%) by studying brain metabolic states by FDG-PET (Ewers et al., 2014). ^11^C-PIB-PET has higher SN (96%), low SP (58%), moderate LR+ (2.3), and LR– (0.07) for the conversion of MCI-AD (Zhang et al., 2014).

One of the main issues of concern of using this proposed algorithm is how to select potential patients to be screened. The selection of patients should be conducted as described in Table 4.

### Universal standardization diagnostic protocols

Standard methods of sample collection, reference standards, universal cut-off values for diagnostic tests, and standard operating procedures (SOP) are urgently needed for most advanced AD biomarkers (CSF core biomarkers, Aβ-PET, FDG-PET, and tau-PET). A significant amount of work has been done by an IWG for a universal standardization of AD diagnostic biomarkers (Mattsson and Zetterberg, 2012; Mattsson et al., 2012, 2013; https://aibl.csiro.au/wp-content/uploads/2014/01/sperling.pdf).

### Estimated costs of AD diagnosis tests

No doubt, there will be several thousand dollar cost for selecting each patient, and more cost would be during longitudinal follow up. An estimated cost of MRI ($1,694–$3,624) is higher than CSF biomarkers (http://www.comparemricost.com/. Study of 10 cities in the USA show that (Orlando, FL Dallas, TX—MRI Testing Facility A MRI and Dallas, TX—MRI Testing Facility B, San Diego, CA, Salt Lake City, UT, Detroit, MI, New York, NY—MRI Testing Facility A, New York, NY—MRI Testing Facility B, Raleigh, NC, Omaha, NE) fMRI costs are even higher than MRI. Specialized bio-informatics personals with expensive equipment are the main reason for the higher cost of modern neuroimaging AD biomarkers. Moderately invasive PET imaging costs $825–$6,800 (http://www.newchoicehealth.com/procedures/pet-scan-brain: National PET Scan Brain Procedure Pricing Summary). Highly invasive CSF biomarker tests are moderately expensive (~$450-$1,000 per test) (Fiandaca et al., 2014; Valcárcel-Nazco et al., 2014), because highly skilled personals are required in specialized medical centers for CSF sample collection (lumbar puncture), three ELISA kits are necessary for (Aβ_42_, tau, and p-tau), and the cost of CSF biomarker tests are much higher than blood-based diagnostic tests. Neuroimaging biomarkers are expensive because modality is a technical sophistication that needs technically trained expert teams of neuroscientists, radiologists, and bioinformatics specialists. A panel of a combination of CSF and neuroimaging biomarkers would increase the predicting power of preclinical AD diagnosis. However, the cost of diagnosis of such AD biomarkers will be much higher. Peripheral AD diagnosis in preclinical AD phase is the best way to decrease this enormous cost.

### The complexity of patient selection for preclinical AD clinical trials

A homogeneous set of patients with preclinical AD certainly would not be available by its own characteristic feature of heterogeneity. Therefore, several subgroups of patients with preclinical AD can be separated according to their initial baseline preclinical AD biomarker values and follow longitudinally for a longer time (5–10 years). Such sub-groups are A. genetic sub-group: Asymptomatic high genetic risk with APOE4 and TOMM40 alleles, B. Neurodegeneration subgroup: Asymptomatic neurodegeneration by brain hypometabolism/tau deposition/atrophy, and C. Asymptomatic high-risk Aβ deposition by Aβ-PET and low CSF Aβ_42_ values. Because of longtime follow-up, preclinical AD longitudinal clinical trials should be expensive. Other drawbacks of such clinical trial are patient withdrawal, interference of comorbidity, and lifestyle changes of patients during the longitudinal long follow-up.

### Ethical challenges

Ethical issues to a conclusion and disclosure of a preclinical AD is very complex. The main ethical concerns for an individual are emotional, social, and economical. Emotional issues are feelings, fears, impact on personal motivation, and behavior to family members. A Metlife Foundation survey in 2011 found Americans middle aged and older (≥55 years) are afraid of AD (31%), more than diabetes, heart disease, or stroke and less than cancer (41%) (Metlife Foundation, 2011). Preclinical AD disclosure may induce anxiety and depression. Social issues, such as the social stigma of future development of AD and withdrawal from social events, are an impact on friendship networks. The economic impact will be enormous to an individual with a preclinical AD diagnosis. Health insurance companies may increase the insurance premium. An individual working in higher cognitive performing jobs, such as pilot, clinical practitioners, and nurses need to be reported to a higher authority. Regulatory authorities should develop suitable guidelines for ethical issues for informing individuals about preclinical AD diagnosis.

Keeping all of these issues in mind, one can look at the positive aspect of the disclosure of preclinical AD. More than 90% responded in a survey that they wanted to adopt a healthier lifestyle if they knew they were at risk of AD (Caselli et al., 2014). Individuals who want to take a positive outlook of preclinical AD diagnosis should be influenced to increase social contact, healthy food, and mental exercises such as numerical problem solving, meditation, and yoga.

### Feasibility and utility of the preclinical AD diagnostic algorithm

The main challenge remains how to choose individuals for preclinical AD diagnostic clinical trials. We proposed four different categories of probable preclinical AD cases and definitive preclinical AD category of pre-symptomatic genetic risk factors (Table 4) to be longitudinally tested in clinical trials by proposed preclinical AD biomarkers. In longitudinal follow-up trials will produce four different categories of biomarkers results (amyloid+ and neurodegenerative+, amyloid+ and neurodegenerative-, amyloid-and neurodegenerative+, amyloid- and neurodegenerative– (Pereira et al., 2017), and cognitive impairment due to AD. Those results will be complied in a comprehensive AD preclinical diagnostic framework presented in Figure 2 to generate correlation of AD converters and AD non-converters with preclinical AD biomarkers.

Although challenges of cost, positive predictive values, and ethical issues are substantial this algorithm will provide a frame work of preclinical AD diagnosis, like lipid profiling for an individual in risk of heart disease. In the future individual with a risk of a preclinical AD from this framework should have suggestion from doctors to follow a healthy lifestyle.

## Author contributions

The author confirms being the sole contributor of this work and approved it for publication.

### Conflict of interest statement

The author declares that the research was conducted in the absence of any commercial or financial relationships that could be construed as a potential conflict of interest.
